# COVID-19-activated SREBP2 disturbs cholesterol biosynthesis and leads to cytokine storm

**DOI:** 10.1038/s41392-020-00292-7

**Published:** 2020-09-03

**Authors:** Wonhwa Lee, June Hong Ahn, Hee Ho Park, Hong Nam Kim, Hyelim Kim, Youngbum Yoo, Hyosoo Shin, Kyung Soo Hong, Jong Geol Jang, Chun Gwon Park, Eun Young Choi, Jong-Sup Bae, Young-Kyo Seo

**Affiliations:** 1 Aging Research Center, Korea Research Institute of Bioscience and Biotechnology, Daejeon, 34141 Republic of Korea; 2 Division of Pulmonology and Allergy, Department of Internal Medicine, College of Medicine, Yeungnam University and Regional Center for Respiratory Diseases, Yeungnam University Medical Center, Daegu, 42415 Republic of Korea; 3 Department of Biotechnology and Bioengineering, Kangwon National University, Chuncheon, Gangwon-do 24341 Republic of Korea; 4 Center for BioMicrosystems, Brain Science Institute, Korea Institute of Science and Technology (KIST), Seoul, 02792 Republic of Korea; 5 Division of Bio-Medical Science and Technology, KIST School, Korea University of Science and Technology, Seoul, 02792 Republic of Korea; 6 College of Pharmacy, Chungnam National University, Daejeon, 34134 Republic of Korea; 7 Department of Biomedical Engineering, SKKU Institute for Convergence, Sungkyunkwan University (SKKU), Suwon, Republic of Korea; 8 Biomedical Institute for Convergence at SKKU (BICS), Sungkyunkwan University, 2066 Seobu-ro, Jangan-gu, Suwon, 16419 Republic of Korea; 9 College of Pharmacy, CMRI, Research Institute of Pharmaceutical Sciences, BK21 Plus KNU Multi-Omics based Creative Drug Research Team, Kyungpook National University, Daegu, 41566 Republic of Korea

**Keywords:** Predictive markers, Infectious diseases

## Abstract

Sterol regulatory element binding protein-2 (SREBP-2) is activated by cytokines or pathogen, such as virus or bacteria, but its association with diminished cholesterol levels in COVID-19 patients is unknown. Here, we evaluated SREBP-2 activation in peripheral blood mononuclear cells of COVID-19 patients and verified the function of SREBP-2 in COVID-19. Intriguingly, we report the first observation of SREBP-2 C-terminal fragment in COVID-19 patients’ blood and propose SREBP-2 C-terminal fragment as an indicator for determining severity. We confirmed that SREBP-2-induced cholesterol biosynthesis was suppressed by Sestrin-1 and PCSK9 expression, while the SREBP-2-induced inflammatory responses was upregulated in COVID-19 ICU patients. Using an infectious disease mouse model, inhibitors of SREBP-2 and NF-κB suppressed cytokine storms caused by viral infection and prevented pulmonary damages. These results collectively suggest that SREBP-2 can serve as an indicator for severity diagnosis and therapeutic target for preventing cytokine storm and lung damage in severe COVID-19 patients.

## Introduction

The recent pandemic caused by unparalleled infectivity of the novel human coronavirus, referred to as severe acute respiratory syndrome coronavirus 2 (SARS-CoV-2), has spread rapidly around the globe causing coronavirus disease 2019 (COVID-19) affecting our health care systems and economies.^1,2^ Phenomenally, symptoms observed in patients are similar to other previously reported viruses, such as severe acute respiratory syndrome (SARS) and Middle East respiratory syndrome (MERS).^3,4^ Many of these patients suffered from pneumonia-associated symptoms, such as acute respiratory distress syndrome (ARDS), cytokine release syndrome (CRS), multiple organ failure (MOF), and sepsis.^5,6^ The term cytokine storm is a physiological reaction in which the immune system causes an uncontrolled and excessive release of proinflammatory signaling molecules called cytokines.^7^ Aggressive inflammatory response with the sudden release of proinflammatory cytokines in large quantities can cause acute respiratory distress syndrome (ARDS) aggravation and widespread tissue damage resulting in multiple organ failure (MOF) and death.^8^ Recently, it has been reported that elevated serum cytokine levels and the severe complications correlate directly with coronavirus disease 2019 (COVID-19).^9^ Mortality in COVID-19 patients has been linked to the overproduction of the proinflammatory cytokines (IFNα, IFNγ, IL-1β, IL-6, IL-12, IL-18, IL-33, TNFα, and TGFβ) and chemokines (CXCL10, CXCL8, CXCL9, CCL2, CCL3, and CCL5) induced by the SARS-CoV-2 virus.^10^

There are no approved therapies nor effective therapeutic agents available for the treatment of COVID-19 and the only available option is supportive treatment.^11,12^ In response to the COVID-19 pandemic, the global effort for research and development of a vaccine is unprecedented in terms of speed and scale. Speed is a paramount factor and there is an indication that vaccines could be available under emergency use by early 2021.^13^ As it still requires time, this raises an important question about the urgent need for the development of effective therapies to lower the mortality rate of symptomatic COVID-19 patients.

Sterol regulatory element binding proteins (SREBPs) were well documented as the basic-helix-loop helix-leucine zipper transcription factors that regulate the gene expressions involved in lipid cholesterol biosynthesis.^14–16^ These family of SREBP transcription factors have been reported to regulate the lipid cholesterol and fatty acid gene expressions^17^ via MAPK activation pathway.^18^ SREBP transcription factor is a critical regulator of lipid biosynthesis and sterol homeostasis in eukaryotes, where in mammals, SREBPs are highly active in the fed state to promote the expression of cholesterogenic and lipogenic genes involved in fat storage.^19^ For example, SREBP-2 has been shown to directly activates autophagy-related genes under sterol starvation condition.^20,21^ Recently, various pathogenic processes have been linked with SREBPs, such as endoplasmic reticulum (ER) stress, inflammation, apoptosis, and autophagy.^22^ Stiffening of diseased tissue has been linked with the SREBP levels as well.^23^

A recently published paper showed that Sestrin1 (SESN1) transcription is regulated by SREBP-2 in cells, demonstrating that SESN1 inhibits cholesterol biosynthesis.^24^ The results showed a significant reduction in plasma cholesterol levels in cholesterol-fed SESN1^+/−^ and SESN1^−/−^ mice but not in control mice (Sesn1^+/+^), indicating that the SESN1 affects plasma cholesterol in a liver-specific manner via regulation of cholesterol biosynthesis. It is reported that SESN1 activates AMP kinase (AMPK) and inhibits rapamycin complex 1 (mTORC1).^25–27^ The recently published data demonstrated that after cholesterol feeding, SESN1^−/−^ mouse livers had significantly reduced phosphorylated AMPK and unchanged mTORC1, indicating that SESN1 functions to repress cholesterol biosynthesis via activating AMP kinase pathway.^24^ However, the exact mechanism of how cholesterol biosynthesis of SREBP-2 and SESN1 is associated with induction of inflammation during infections remain elusive.

It is well-understood that cytokines, such as type I interferon (IFN) protects against viruses via suppressive effect on inflammation. It was reported that hydroxylated form of cholesterol is a critical mediator in the negative-feedback pathway of IFN signaling on IL-1 family cytokine production and inflammasome activity.^28^ In addition, growing evidences show that hydroxycholesterols play an important role as regulators of immune function, demonstrating their roles are closely related to alteration of cholesterol content in plasma membrane that can have antiviral, anti-inflammatory, and proinflammatory effects.^29^ Hydroxycholesterol functions as an immune cell guidance signal by engaging the EBI2 receptor, a member of the G protein-coupled receptor, that it is required for innate and adaptive immunity. Specifically, cytokine production such as IFN induced by viral and bacterial infection along with toll-like receptor (TLR) signaling induces active nuclear translocation of hydrocycholesterol from ER, thus secretion of IL-6, IL-8, and M-CSF cytokines. This is consistent with observations that cholesterol consumption leads to increase in inflammation and thus activates SREBP-2. Although the SREBP-2 is known to be a transcription factor for lipid synthesis, we found that the level of cholesterol maintained in a low level in COVID-19 patients, and even though the expression level of SREBP-2 is increased in plasma of COVID-19 patients.

A recent report has shown evidence of biological crosstalk between SREBP-2 and NF-κB.^30^ It is known that that the cells secrete lipid and cholesterol to inactivate viruses.^31–34^ Recently, it has been reported that the activity of SREBP-2 increases as the concentration of cholesterol in the cell decreases.^33,35^ Based on these findings, we hypothesized that the activation of cholesterol biosynthesis is as a result of decrease in cellular cholesterol level and subsequent activation of SREBP-2. It is reported that SREBP-2-mediated biosynthesis of cholesterol is involved in the exocytosis process of SARS-CoV2, which explains its role in virus budding and envelop.^31^

Herein, we propose that the activated SREBP-2 and NF-κB can cause vascular and organ damage, and believe that our study has proven its potential as a therapeutic target for the treatment of viral infections. We hypothesized that in infectious diseases, the role of mature SREBP-2 as an inflammatory transcription factor and its role as a diagnostic marker of SREBP-2 C-term. In this study, we traced SREBP-2 in COVID-19 patients and demonstrated for the first time that C-term fragment of SREBP-2 is found in the blood of COVID-19 patients. Upon SARS-CoV-2 viral infection, SREBP-2 C-term fragment serves as an endotoxin, causing cytokine storm in COVID-19 septic patients. We monitored the level of SREBP-2 C-term fragment in these patients and identified the increase correlated with inflammatory cytokine release and vascular disruption. Knockdown of the SREBP-2 rescued LPS-induced inflammatory cytokine levels. We showed that an intravenous administration of short hairpin SREBP-2 (shSREBP2) alleviate lung damage and improve survival in a sepsis model. This is the first evidence that show secretion of SREBP-2 C-term in severe COVID-19 patients for the first time and propose as a biomarker for determination of the severity of the COVID-19. It is anticipated that targeting ubiquitously expressed transcription factors, SREBP-2 and NF-κB may hamper and slow down the progression of the symptom and reduce the severity of the disease. It is of vital importance to suppress the expression and secretion cytokines in severe COVID-19 patients.^9^ Antiviral drug such as remdesivir do not improve mortality in severe COVID-19, but it has been reported that anti-inflammatory drug such as dexamethasone ameliorates mortality.^36,37^ Steroid-based drugs have shown improvement in the mortality rate of COVID-19 severe patients, therefore regulation of uncontrolled inflammatory responses is a promising treatment strategy. These finding indicate targeting SREBP-2 C-term fragment as a potential therapeutic strategy for SARS-CoV-2 that could play as a key factor for the treatment of severe COVID-19 patients with sepsis.

## Results

### SREBP-2 was highly activated in COVID-19 patients’ PBMCs and affected subsequent cytotoxic effects on PBMCs

**Fig. 1:**
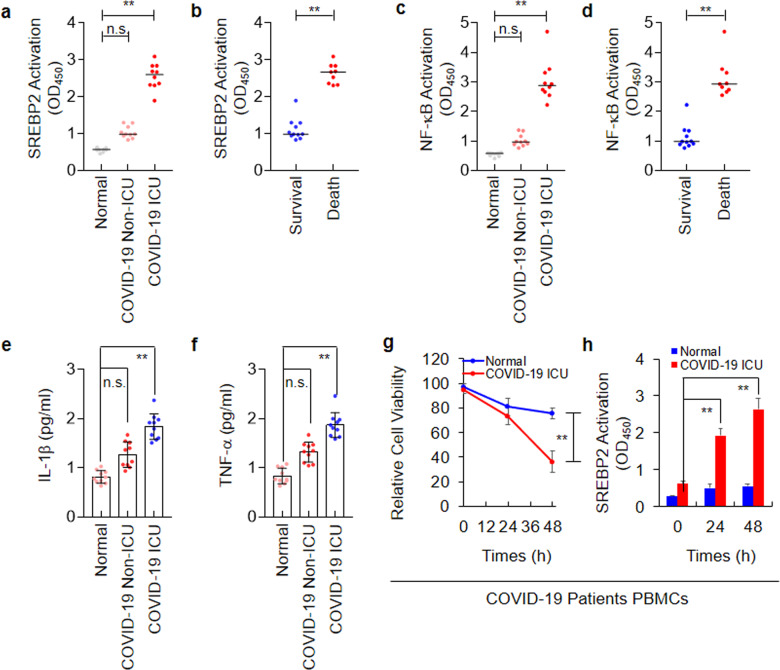
Analysis of patients’ blood revealed the SREBP-2 as a severity diagnostic marker for COVID-19. **a** Activation level of SREBP-2 with respect to the severity of COVID-19 (**p < 0.01). **b** Activation level of SREBP-2 in survival and deceased patients of COVID-19 (**p < 0.01). **c** Activation level of NF-κB with respect to the severity of COVID-19 (**p < 0.01). **d** Activation level of NF-κB in survival and deceased patients of COVID-19 (**p < 0.01). **e** Level of IL-1β in the COVID-19 patients’ plasma (**p < 0.01). **f** Level of TNF-α in the COVID-19 patients’ plasma (**p < 0.01). **g** Relative viability of PBMCs obtained from COVID-19 patients with respect to the culture time in vitro (**p < 0.01). **h** Activation level of SREBP-2 in PBMCs obtained from COVID-19 patients (**p < 0.01, n.s. not significant)

In the blood of COVID-19 patients, the levels of total cholesterol (Ch), high-density lipoprotein (HDL)-Ch, and low-density lipoprotein (LDL)-Ch were lower compared to normal case, and the level was lower in intensive care unit (ICU) patient than nonICU patients (Supplementary Table S1). There were no notable comorbidities in each group. According to the analysis, the SREBP-2 activity was increased as the severity of COVID-19 was increased from nonICU to ICU (Fig. 1a), which is inverse correlation with the trend of cholesterol level (Supplementary Table S1). The activation level of SREBP-2 was higher in deceased patients than survival case (Fig. 1b), suggesting the SREBP-2 as an indicator for severity of COVID-19. Furthermore, nuclear factor (NF)-κB, which is known as a crosstalk molecule of SREBP-2,^30^ was exhibited similar increasing trend as the severity of COVID-19 increases (Fig. 1c, d). Production of inflammatory cytokines such as interleukin (IL)-1β and tumor necrosis factor (TNF)-α by SREBP-2 or NF-κB were also increased as the severity of COVID-19 increases (Fig. 1e, f). To determine whether SREBP-2 activation is as a direct cause of SARS-CoV-2 or SARS-CoV-2 viral proteins, we examined activation of SREBP-2 by treating recombinant SARS-CoV-2 spike RBD protein to high ACE2-expressing cells, HUVECs (Supplementary Fig. 1). The results showed no significant difference in the SREBP-2 level. According to qRT-PCR, SREBF2 mRNA as increased in COVID-19 patients in a severity-dependent manner, and the level of sestrin 1 (SESN1) and proprotein convertase subtilisin/kexin type 9 (PCSK9), which are known to regulate the lipid biosynthesis, also showed similar increasing trend as the severity of COVID-19 increases (Supplementary Fig. 2). On the other hand, the mRNA levels of 3-hydroxy-3-methylglutaryl-CoA reductase (HMGCR), an enzyme acting in the upstream of cholesterol synthesis,^38^ and low-density lipoprotein receptor (LDLR) were not changed irrespective of the severity of COVID-19 (Supplementary Fig. 2). These results suggest that COVID-19 infection inhibits the direct synthesis pathway of cholesterol by SREBP-2, while increasing its activity as an inflammatory transcription factor. When the COVID-19 patients’ PBMCs were cultured in vitro, the viability of PBMCs isolated from the blood of COVID-19 ICU and acute respiratory distress syndrome (ARDS) patients was decreased rapidly as time compared to normal case (Fig. 1g). Besides, the activation level of SREBP-2 was increased in cultured PBMCs as time, implying the activation of SREBP-2 is transient (Fig. 1h).

### The level of SREBP-2 C-term reflect the severity of COVID-19

**Fig. 2:**
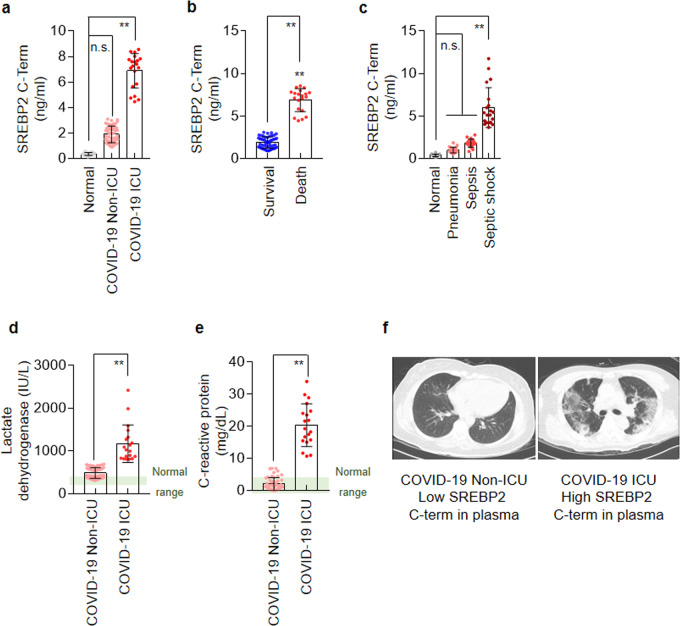
SREBP-2 C-term reflects the severity of infectious diseases, and thus can be used as a diagnostic marker. **a** Level of SREBP-2 C-term in COVID-19 patients’ plasma (**p < 0.01). **b** Level of SREBP-2 C-term in survival and deceased patients’ plasma (**p < 0.01). **c** Level of SREBP-2 C-term in the plasma of pneumonia, sepsis, and septic shock patients (**p < 0.01). **d**, **e** Level of lactate dehydrogenase (LDH) (**d**) and C-reactive protein (CRP) (**e**) in nonICU and ICU COVID-19 patients (**p < 0.01). **f** Computed tomography (CT) images of COVID-19 patients’ lung depending on the level of SREBP-2 C-term (**p < 0.01, n.s. not significant)

Previously, the role of SREBP-2 N-term was largely demonstrated by many researches. Upon the cleavage of SREBP-2 N-term and C-term by S1P and S2P, the N-term was translocated to the nucleus and ultimately regulated the synthesis of cholesterol.^14^ However, the role of SREBP-2 C-term has not been reported yet. Here, we hypothesized that the SREBP-2 C-term should be secreted in the blood of COVID-19 patients in response to the degree to which the SREBP-2 was activated. Especially, in the severe cases of COVID-19 patients including ICU and deceased cases, the level of SREBP-2 C-term was dramatically increased (Fig. 2a, b). The SREBP-2 C-term was also secreted in severe sepsis case (septic shock) (Fig. 2c), implying the availability of SREBP-2 C-term as a general indicator for infectious diseases. This idea is further supported by the increased level of lactate dehydrogenase (LDH) and C-reactive protein (CRP) in the blood of COVID-19 patients (Fig. 2d, e). This high level of SREBP-2 C-term is closely related to the hyper-inflammation in lung tissue of COVID-19 patients. The computed tomography (CT) image of ICU patient with high SREBP-2 C-term (right panel of Fig. 2f) in plasma displayed severe lung inflammation than nonICU patient with low SREBP-2 C-term level (left panel of Fig. 2f). To the best of authors’ knowledge, the detection of SREBP-2 C-term and the demonstration of correlation with infectious diseases has not reported yet.

### The inhibition of NF-κB and SREBP-2 suppresses the SREBP-2 activation and inflammatory cytokine production

**Fig. 3:**
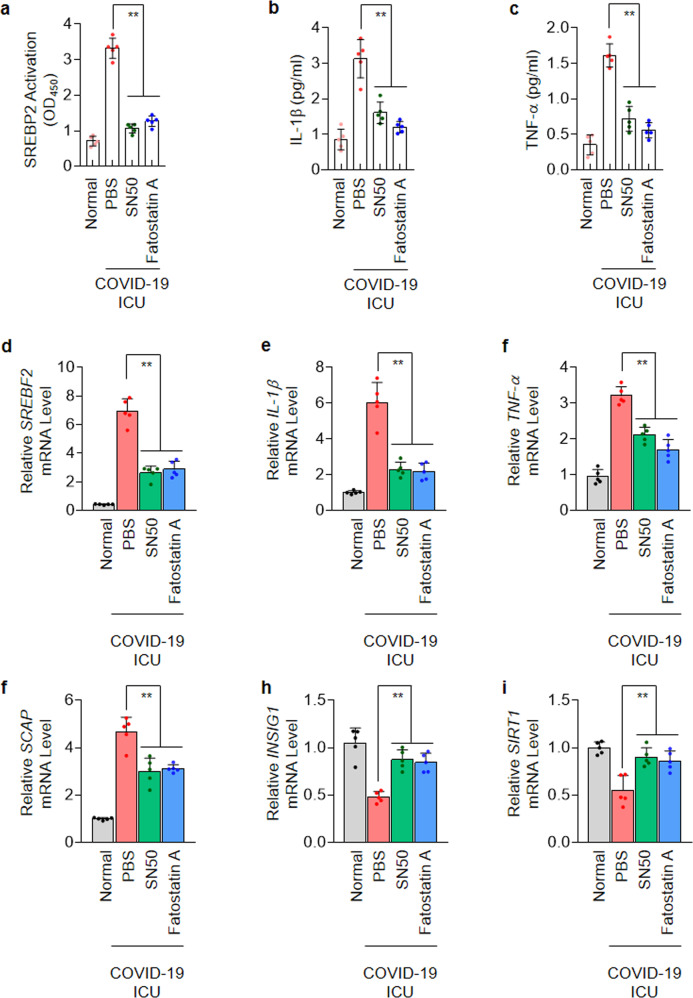
Inhibition of SREBP-2 and NF-κB prevents the inflammatory cytokines and mRNA levels related to SREBP-2. **a** Activation level of SREBP-2 in COVID-19 ICU patients’s PBMCs by the treatment of NF-κB inhibitor (SN50) and SREBP-2 inhibitor (Fatostatin A) (**p < 0.01). **b**, **c** Level of IL-1β (**b**) and TNF-α (**c**) in COVID-19 ICU patients’s PBMCs by the treatment of SN50 and Fatostatin A (**p < 0.01). **d**–**i** Changed level of mRNA after the treatment of SN50 and Fatostatin A. **d** SREBF2, **e** IL-1β, **f** TNF-α, **g** SCAP, **h** INSIG1, and **i** SIRT1 (**p < 0.01)

We confirmed the possibility of restoration of SREBP-2 level and subsequent cytokine storm by direct pharmacological inhibition of SREBP-2 with Fatostatin A (a SREBP-2 processing inhibitor) and by regulating upstream NF-κB signaling with SN50 (a NF-κB signaling inhibitor). The treatment of SN50 and Fatostatin A suppressed the activation level of SREBP-2 in PBMCs of COVID-19 ICU patients (Fig. 3a). Further, the pharmacological inhibition also suppressed the inflammatory cytokine production such as IL-1β and TNF-α (Fig. 3b, c). qRT-PCR analysis also confirmed the suppression of relevant mRNAs including SREBF2, IL-1β, and TNF-α (Fig. 3d, f). The mRNA level of SCAP (SREBP cleavage-activating protein) and INSIG1 (insulin-induced gene 1, a negative regulator of SREBP-2) were rescued by the pharmacological inhibition of SREBP-2 and NF-κB (Fig. 3g, h). The mRNA expression of SIRT1 as regulator of nucleus SREBP-2 stability was also rescued as a result of pharmacological inhibition. Although the NF-κB activation could be rescued directly by SN50 treatment, it could not be fully rescued by Fatostatin A, showing that the NF-κB is an upstream regulator of SREBP-2 (Supplementary Fig. 3).

### SREBP-2 C-term is the indicator of dysregulated vasculature and can be protected by pharmacological inhibition or knockdown of SREBP-2

**Fig. 4:**
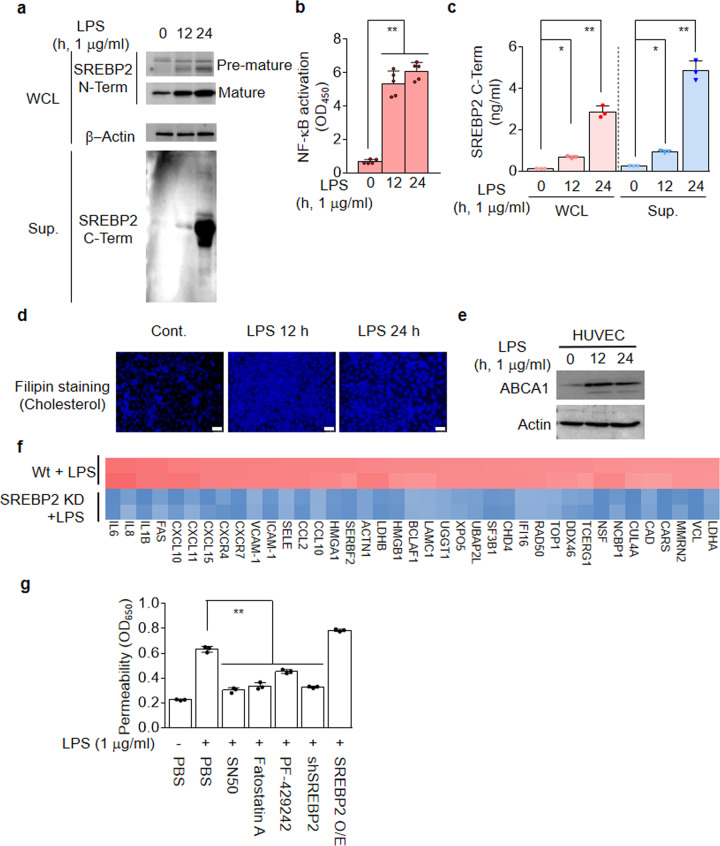
SREBP-2 activation is critical for the vascular inflammatory responses via cholesterol release and cytokine expression. **a** Western blot analysis of N-term and C-term of SREBP-2 in whole cell lysate (WCL) and supernatant (Sup.) after the LPS stimulation (1 μg/ml). **b** Time-dependent NF-κB activation after the LPS stimulation (1 μg/ml) (**p < 0.01). **c** Time-dependent secretion of SREBP-2 C-term in WCL and Sup. after the LPS stimulation (1 μg/ml) (**p < 0.01). **d** Filipin staining after the LPS stimulation (Scale bar: 200 μm). **e** Western blot analysis of ATP-binding cassette transporter (ABCA1) in human umbilical vein endothelial cell (HUVEC) after the LPS stimulation (1 μg/ml). **f** Effect of SREBP-2 knockdown in the suppression of cytokine production. **g** Transendothelial permeability of HUVEC after the inhibition or overexpression (O/E) of relevant signaling (*p < 0.05, **p < 0.01)

To demonstrate the different translocation target of N-term and C-term of SREBP-2, we performed western blot analysis. Upon the exposure to lipopolysaccharide (LPS), the expression of SREBP-2 N-term is transiently increased as time in whole cell lysate (WCL) (Fig. 4a) of HUVEC. On the other hand, the SREBP-2 C-term was highly expressed in supernatant at the late-stage of LPS stimulation (24 h) (Fig. 4a). Similarly, in other cell types such as HEK293 and human umbilical vein endothelial cell (HUVEC), the SREBP-2 N-term was detected in cell lysate, but not observed in culture media (Supplementary Fig. 4). NF-κB activation by LPS was elevated early, unlike SREBP-2 as late mediator,^38^ which is interpreted to mediate serious lung injury by crosstalk between NF-κB and SREBP-2 (Fig. 3b).^30^ The SREBP-2 C-term was detectable in both WCL and supernatant, but the level was higher in supernatant (Fig. 4a). The notable difference between N-term and C-term of SREBP-2 was the increasing rate with respect to the simulation time. This was consistent with time dependent NF-κB activation upon LPS treatment (Fig. 4b). In contrast to the monotonic increase of SREBP-2 N-term, the SREBP-2 C-term was dramatically increased on 24 h after LPS stimulation (Fig. 4c).

The duration of LPS stimulation induced different consequence in cholesterol metabolism. Filipin staining which visualizes the intracellular cholesterol showed that cholesterol was accumulated in HUVEC after the 12 h of LPS stimulation (Fig. 4d). However, the cholesterol level was decreased 24 h after the LPS stimulation (Fig. 4d). The western blot analysis of ATP-binding cassette transporter (ABCA1), also known as the cholesterol efflux regulatory protein (CERP), showed the decreased expression on 24 h than 12 h (Fig. 4e).

In the HUVEC, the LPS stimulation induces upregulated secretion of inflammatory cytokines (upper panel of Fig. 4f). However, the genetic ablation of SREBP-2 could suppress the cytokine storm even after the LPS stimulation (lower panel of Fig. 4f). The pharmacological inhibition of NF-κB, SREBP-2, and S1P (PF-429242) and short hairpin RNA (shRNA) of SREBP-2 suppressed the vascular barrier disruption even under LPS stimulation (Fig. 4g). SREBP-2-overexpressed (SREBP-2 O/E) HUVECs were more severely damaged by LPS (Fig. 4g).

### SREBP-2 inhibitor improves mortality of infectious diseases mouse model

The pharmacological inhibition of NF-κB signaling and SREBP-2 was valid in the PBMCs of COVID-19 ICU patients as well. To validate the effect of SREBP-2 inhibition as a therapeutic target on infectious diseases with septic shock, we utilized the cecal ligation and puncture (CLP) model for recapitulating pathophysiology of sepsis.^39^ The exogenous incorporation of Fatostatin A and SN50 recovered the mRNA levels of Srebp2, Sens1, and Pcsk9 to lower range (Supplementary Fig. 5a–c). Since the mRNA levels of HMGCR and LDLR were not different in response to COVID-19 infection (Supplementary Fig. 2), the pharmacological inhibition did not induce any change in CLP-operated mice (Supplementary Fig. 5d, e).

**Fig. 5:**
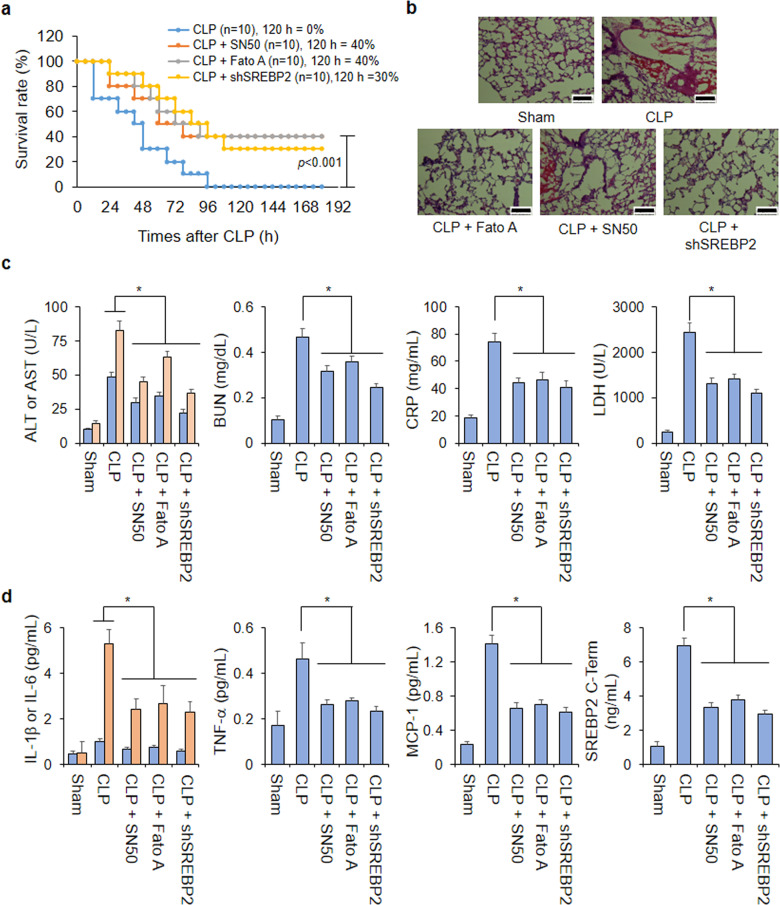
Pharmacological inhibition of NF-κB signaling and SREBP-2 prevent lung tissue damage and rescue survival rate in infectious disease mouse model. **a** Survival rate of sepsis mouse model after the cecal ligation and puncture (CLP). The inhibition of NF-κB signaling (SN50) and SREBP-2 (Fatostatin A and shSREBP-2) rescued the survival rate to 30–40%. **b** Histology of mouse lung tissue with or without the pharmacological inhibition (Scale bar: 100 μm). **c** Levels of tissue damage markers with or without the pharmacological inhibition. Liver damage marker: aminotransferase (AST) and alkaline phosphatase (ALP). Kidney damage marker: blood urea nitrogen (BUN). Inflammation marker: C-reactive protein (CRP) and lactate dehydrogenase (LDH). **d** Levels of cytokines (IL-1β, IL-6, and TNF-α), chemokine (monocyte chemoattractant protein-1 (MCP-1)), and SREBP-2 C-term. (*p < 0.05)

For the SREBP-2 shRNA induced knockdown (KD), SREBP-2 shRNA was delivered via intravenous (i.v.) injection daily the CLP was performed (Supplementary Fig. 6). We confirmed that the shSREBP2 reduced the mRNA level of SREBP-2 in mouse plasma (Supplementary Fig. 6). The inhibition of NF-κB signaling with SN50 (40%) and SREBP-2 with Fatostatin A (40%) and SREBP-2 KD (30%) rescued the survival rate of mouse model, respectively (Fig. 5a). Histology of mouse lung tissue also confirmed that the inhibition of NF-κB signaling and SREBP-2 prohibited the lung failure (Fig. 5b). Various tissue damage markers were also decreased in mouse plasma as a result of pharmacological inhibition: liver—alanine aminotransferase (ALT) and aminotransferase (AST), Kidney—blood urea nitrogen (BUN), Inflammation—C-reactive protein (CRP) and lactate dehydrogenase (LDH) (Fig. 5c). The inflammatory cytokines including 1L-1β, IL-6, and TNF-α, the chemokine monocyte chemoattractant protein 1 (MCP1), and SREBP-2 C-term were also maintained in a low level in mouse plasma (Fig. 5d). The mRNA levels of nitrite/nitrate oxide (NOX), which is an indicator of sepsis, NLR family pyrin domain containing 3 (NLRP3) inflammasome, which plays a crucial role in innate immunity and inflammation,^40^ Il-1β, vascular cell adhesion molecule 1 (VCAM-1), intercellular adhesion molecule-1 (ICAM-1), and SREBP-2 in mouse lung tissue were also maintained in low level in response to the inhibition of NF-κB signaling and SREBP-2 (Supplementary Fig. 7). The experimental results in both COVID-19 patients’ PBMCs and mouse model collectively suggest that the inhibition of NF-κB signaling and SREBP-2 may play a pivotal role as therapeutics for infectious diseases.

## Discussion

Recently, the decreased level of total, HDL, and LDL was reported in the COVID-19 patients in Wenzhou, China.^41^ In our study, the lower level of the total, HDL and LDL were also observed in COVID-19 patients in Daegu, Korea, and the level was further decreased as the symptoms worsens. Upon the viral infection, as well as in response to the entry of endotoxin, such as LPS, the cholesterol tends to inactivate their biological toxicity through the selective interaction.^32,42^ It is well known that decreased cholesterol level in serum and cells drives the dissociation of SCAP-SREBP-2 complex from INSIGs and subsequently the SCAP-SREBP-2 complex translocates to Golgi.^14^ The SREBPs was cleaved into N-term and C-term by enzymes including S1P and S2P, and the SREBP-2 N-term translocates to the nucleus and activates transcription of lipid synthesis. Similar, it was shown that a cross-talk between TLR4-MyD88-NF-κB and SCAP-SREBP-2 pathways mediate macrophage foam cell formation and thereby inducing inflammatory responses.^30^ Intracellular cholesterol content was observed and it was shown that LPS increases both gene and protein expression levels of LDLR, HMG-CoAR, SCAP, and SREBP-2, leading to translocation of SREBP to organelles.

However, according to the analysis, the mRNA expression levels of SESN1 and PCSK9, that suppress the lipid biosynthesis, were increased in the PBMCs of COVID-19 patients. Cholesterol biosynthesis is inhibited by the expression of SESN-1 and PCSK9, and is regulated by transcription factor and NRLP3 inflammasome.^24,43^ A recent study has confirmed that cholesterol biosynthesis is increased by the activity of SREBP-2; but at the same time, SESN-1 and PCSK9 are expressed and inhibitory results were demonstrated.^24^ In severe COVID-19 patients, the biosynthesis of cholesterol such as intracellular HDL needs to be promoted and then subsequent homeostasis takes place as a result of the increase in SREBP-2 activity.

As a result of upregulation of lipid synthesis-suppressing genes, cells do not synthesize cholesterol. Instead, in the COVID-19 infection and sepsis cases, SREBP-2 regulates the production of IL-1β and TNF-α, which is well displayed in Fig. 1e, f. Further, we demonstrated that the SREBP-2 is also upregulated as a consequence of NF-κB. This supports a recent report of a biological crosstalk between SREBP2 and NF-κB.^30,44,45^ When cells are infected, they secrete lipid and cholesterol to inactivate the viruses.^32,34,46^ Recently, it has been reported that, SREBP2, which regulates cholesterol biosynthesis, act as a signaling hub for inflammation and cholesterol metabolism.^33,35^ Based on these findings, we can deduce that the activation of cholesterol biosynthesis is as a result of decrease in cellular cholesterol level and subsequent activation of SREBP-2. SREBP-2-mediated biosynthesis of cholesterol is involved in the exocytosis process of SARS-CoV2, which explains its role in virus budding and envelop.^31,47,48^ For the inhibition of this process, Statin-based drugs have been suggested as therapeutic agents.^31^ These mechanisms collectively suggest that various responses caused by SARS-CoV-2 infection activates SREBP-2, as demonstrated in Fig. 1.

We have noticed changes in the circulating levels of HDL and LDL in COVID-19 patients and assume they could serve as markers for prediction of the severity of the disease, given sufficient database is attained and statistical significance is found. A standard need to be established based on the cholesterol level in infected patients with respect to body mass index (BMI). Many researchers have already studied the connection between lipid metabolism and SARS-CoV-2,^49^ and provided that these results are databased in the future we may be able to suggest HDL and LDL as a severity marker of COVID-19.

ARDS and septic shock are the main symptoms of severe COVID-19 patients, and are the leading causes of death.^50,51^ In particular, we monitored high levels of activation of the selected treatment targets, SREBP-2 and NF-κB (Fig. 1). The CLP model that we used in this study is a polymicrobial septic mouse model. It is widely acknowledged that cecal ligation and puncture (CLP) or lipopolysaccharide (LPS) induces systemic inflammation and overproduction of cytokines in C57BL/6N mice.^52^ We noticed that the bacterial infection induced not only cytokine storm, but also increased the levels of activated SREBP-2 and NF-κB, and mRNA levels of other related proteins (Fig. 5). SREBP-2-activated and NF-κB-activated PBMCs of COVID-19 patients, together with CLP mouse model were used in this study to evaluate the therapeutic efficacy of the inhibitors. We have used the LPS cellular model because it was previously verified that NF-κB and SREBP-2 are activated by LPS.^30,44,45^ As for the cellular model, it is important to demonstrate lung damage and long-term damage caused by the viral infection by observing the vascular integrity. HUVEC was used because the correlation between SARS-CoV-2 and endothelial disruption is important. As for HEK293, it was used in evaluation to assess the activity of SREBP-2 via genetic modification.

In addition, since the SREBP-2 is ubiquitously in many cell types, its inhibition can serve as an anticipative therapeutic strategy for infectious diseases. As shown in our pharmacological inhibition of SREBP-2 and NF-κB using inhibitors and shRNA, the early-stage inhibition of them could indeed suppress the tissue damage, overproduction of inflammatory cytokines, and death.

It is noted that we found the SREBP-2 C-term as a diagnostic marker for severity of infectious diseases. In spite of in-depth studies on SREBP-2 N-term, the roles and trajectories of enzyme-dissociated SREBP-2 C-term has not been reported. In our study, to the best of our knowledge, we demonstrate the secretion of SREBP-2 C-term for the first time. According to our study, SREBP-2 C-term is found as a secreted form in the patients’ plasma and culture media, and also detected in PBMCs. These results showed that SREBP-2 C-term is released out of cells and circulate plasma. This extracellular secretion of SREBP-2 C-term can be used for the diagnostic marker.

**Fig. 6:**
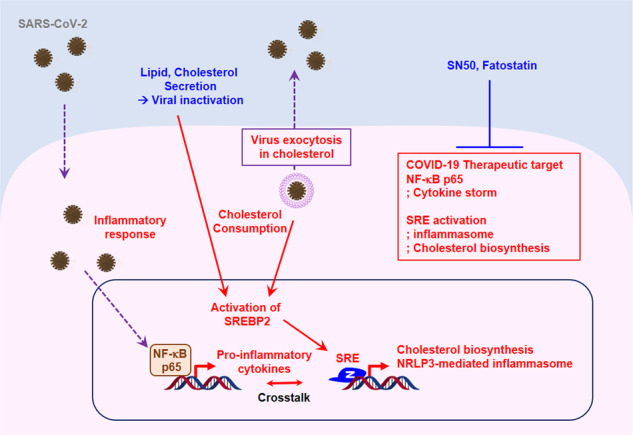
The mechanism of activation of SREBP-2 in COVID-19 patients. The activity of SREBP-2 is regulated by cholesterol consumption and crosstalk between NF-κB from various inflammatory response processes induced by SARS-CoV-2 virus infection

Here, inspired by finding of SREBP-2 C-term fragment in blood, we hypothesized that the underlying infection with SREBP-2 mechanism involves (1) signal transduction of cytokine storm and activating vascular inflammatory responses, (2) secretion of cholesterol complex with pathogens, (3) reduction of cytoplasmic cholesterol and SREBP-2 activation by S1P and S2P cleavage, (4) SREBP-2 N-term fragment translocates to nucleus and activation of cholesterol biosynthesis and inflammation, (5) leading to SREBP-2 C-term fragment secretion alone or as a vesicle (granule) along with SCAP in the membrane (Fig. 6). Whether generated SREBP-2 C-term fragment undergo lysis, recycle, or exocytosis (secretion), and in what particular form needs to be further elucidated. Nonetheless, observation of SREBP-2 C-term fragment in septic patient blood demonstrate that it can be used as a septic biomarker.

This discovery of SREBP-2 C-term fragment in patient’s blood considered a suitable biomarker candidate and potentially a can be considered as a theragnostic marker for determining the severity and treatment target for severe COVID-19 patients. These results collectively suggest that SREBP-2 C-term fragment may be a key therapeutic target for preventing cytokine storm and organ damages in severe COVID-19 patients with sepsis. Strategies against SREBP-2 N-term fragment deacetylation and SREBP-2 C-term fragment secretion and their associated cellular cholesterol biosynthesis and inflammatory pathways may have preventive and therapeutic potentials in organ injury. The major drawback of current markers of sepsis is that they cannot determine the severity of the disease, whereas higher accuracy and precision is anticipated when comparison is made between the cholesterol and SREBP-2 C-term levels. The advantages of SREBP-2 over the current biomarker of sepsis is that it can determine the severity of the disease as well as higher indication of the positive result can be obtained. We anticipate new therapeutic possibilities based on targeting and modulating SREBP-2 N-term fragments and/or the changing SREBP-2-dependent epigenetics in cells.

## Materials and methods

### Plasma sample

Whole blood was collected from patients admitted at Yeungnam University Medical Center after they were diagnosed with the SARS-CoV-2 infection at a public health center in Daegu, Republic of Korea. Patients with COVID-19 sepsis were defined using criteria provided by the Sepsis Consensus Conference Committee.^53^ Pneumonia and septic shock patients were collected from patients admitted at Yeungnam University Medical Center. Healthy volunteers (*n* = 20) were used as controls. In this study, 50 nonICU and 20 ICU patient samples were used. Clinical data were collected for all the patients (Supplementary Table 1). Plasma samples were prepared by centrifugation at 2000 × *g* for 5 min within 12 h after whole blood collection. The human study protocol was approved by the Institutional Review Board of Yeungnam University Hospital at Daegu in Korea (YUH 2018-05-022, 2020–03–057, 2020–05–031–001).

### Total cholesterol, HDL-cholesterol, and LDL-cholesterol in patients blood

The total cholesterol, HDL-cholesterol, and LDL-cholesterol dataset were analyzed using the modular DPE system (Roche Diagnostics, Basel, Switzerland).^54^

### PBMC isolation and culture

Samples from healthy, SARS-CoV-2 pneumonia patients, or discharged patients were obtained from Yeungnam University Medical Center. The relevant local Institutional Review Boards and Ethics Committees approved the study. Heparinized blood samples were used fresh within 4 h, and peripheral blood mononuclear cells (PBMCs) were separated from blood using Ficoll–Hypaquek or NycoPrepk according to the manufacturer’s recommendations. Following this, more refined PBMCs were obtained via MACSprep™ PBMC Isolation Kit and cultured in RPMI-1640 with 1 mM Sodium pyruvate, 2 mM l-glutamine, 4.5 mg/l glucose, 10 mM HEPES and 2 mg/l sodium bicarbonate.

### SREBP-2 transcriptional activity assays

The transcriptional activities of SREBP-2 were determined by the ELISA method using kits from Abcam (ab133111, Abcam) following manufacturer’s protocol. Briefly, nuclear homogenate equivalent to 30 μg of the protein content was added to each of the wells of the 96-well plate containing the double-stranded DNA sequence harboring the consensus SREBP-binding sequence (sterol regulatory element, SRE) coated onto the wells. The nuclear extract was allowed to hybridize with the coated double-stranded DNA sequence harboring the consensus SRE in the plate overnight at 4 °C. The activated SREBP transcription factor complex was detected by addition of a specific primary antibody directed against SREBP-2 and a secondary antibody conjugated to HRP added to provide a sensitive colorimetric readout at 450 nm.

### NF-κB transcriptional activity assays

Preparation of nuclear extracts and TransAM assays were performed as previously described.^55^ The activity of individual NF-κB subunits was determined using an ELISA-based NF-κB Family Transcription Factor Assay Kit (43296; Active Motif, Carlsbad, CA, USA). Briefly, nuclear extracts (2 μg) were incubated in a 96-well plate, which was coated with NF-κB consensus oligonucleotides. The captured complexes were incubated with specific NF-κB primary Abs and subsequently detected using HRP-conjugated secondary Abs included with the kit. Finally, the optical density (OD) at 450 nm was measured using a Tecan Spark microplate reader (Tecan, Austria GmbH, Austria).

### SREBP-2 C-term ELISA

We performed competitive ELISA using antibodies that recognize the SREBP-2 C-term. SREBP-2 C-term (a.a.639–1031) proteins were 2 μg/100 μl diluted and coated onto Nunc-Immuno™ MicroWell™ 96-well plates and incubated overnight at 4 °C. Prior to use, the plates were washed 3 times with PBST and blocked with 3% BSA in PBS for 30 min at 37 °C. Primary anti-SREBP2 C-term (a.a.801–900) polyclonal antibody (ab194667, abcam, Cambridge, United Kingdom, 1:2000 dilution, 100 μl) and plasma sample (20 μg/100 μl) was pre-incubated for 1 h at 37 °C and then the pre-incubated sample were transferred to peptide-coated plate and incubated for 1 h at 37 °C. The plate was washed five times with PBST. Secondary antimouse antibody (#7076, Cell Signaling Technology, Beverly, MA, 1:5000 dilution, 100 μl) was incubated for 30 min at 37 °C and then the plate was washed five times with PBST. The washed plate was treated with TMB ELISA substrate 100 μl/well for 10 min 37 °C and then Stop Solution 100 μl/well was added. The detection was performed at 450 nm by microplate reader (TECAN Männedorf, Switzerland).

### Statistical analysis

All experiments were performed independently at least three times. Statistically significant differences were determined using unpaired *t*-test. Graphprism 7 was used for statistical analyses. Data are reported as mean ± SEM with significance set at *p* < 0.05. *p*-values for each experiment is provided in the figure legends.

## Supplementary information

SUPPLEMENTAL MATERIAL

## Data Availability

The datasets used and/or analyzed to support the findings of this study are available in this paper or the Supplementary Information. Any other raw data that support the findings of this study are available from the corresponding author upon reasonable request.
