# Supplementary material for: COVID-19-activated SREBP2 disturbs cholesterol biosynthesis and leads to cytokine storm
Source: Signal Transduct Target Ther. 2020 Sep 3;5:186. doi: 10.1038/s41392-020-00292-7 (PMC7471497; doi:10.1038/s41392-020-00292-7)
Supplement: Supplementary file 1 — SUPPLEMENTAL MATERIAL [file 41392_2020_292_MOESM1_ESM.docx]

Supplementary Materials for

**COVID-19-activated SREBP2 disturbs cholesterol biosynthesis and leads to cytokine storm**

Wonhwa Lee1,*, June Hong Ahn2,*, Hee Ho Park3,*, Hong Nam Kim4,5,*, Hyelim Kim6, Youngbum Yoo1, Hyosoo Shin1,6, Kyung Soo Hong7, Jong Geol Jang7, Chun Gwon Park7, 8, Eun Young Choi2, #, Jong-Sup Bae9, #, Young-Kyo Seo1, #

^1^Aging Research Center, Korea Research Institute of Bioscience and Biotechnology, Daejeon 34141 Republic of Korea

^2^Division of Pulmonology and Allergy, Department of Internal Medicine, College of Medicine, Yeungnam University and Regional Center for Respiratory Diseases, Yeungnam University Medical Center, Daegu 42415 Republic of Korea

^3^Department of Biotechnology and Bioengineering, Kangwon National University, Chuncheon, Gangwon-do 24341, Republic of Korea

^4^Center for BioMicrosystems, Brain Science Institute, Korea Institute of Science and Technology (KIST), Seoul 02792, Republic of Korea

^5^Division of Bio-Medical Science and Technology, KIST School, Korea University of Science and Technology, Seoul 02792, Republic of Korea

^6^College of Pharmacy, Chungnam National University, Daejeon 34134, Republic of Korea

^7^Department of Biomedical Engineering, SKKU Institute for Convergence, Sungkyunkwan University (SKKU), Suwon, Republic of Korea

^8^Biomedical Institute for Convergence at SKKU (BICS), Sungkyunkwan University, 2066 Seobu-ro, Jangan-gu, Suwon 16419, Republic of Korea

^9^College of Pharmacy, CMRI, Research Institute of Pharmaceutical Sciences, BK21 Plus KNU Multi-Omics based Creative Drug Research Team, Kyungpook National University, Daegu 41566, Republic of Korea

*These authors contributed equally: Wonhwa Lee, June Hong Ahn, Hee Ho Park, Hong Nam Kim

#Co-corresponding author: E. Y. Choi(letact@yu.ac.kr), J.-S. Bae (baejs@knu.ac.kr), and Y.K. Seo (ykseo@kribb.re.kr)

**This PDF file includes:**

Materials and Methods

Figures. S1 to S8

Table S1 to S2

**Other Supplementary Materials for this manuscript include the following:**

Materials and Methods

Figures S1 to S8

Tables S1 to S2

**Materials and Methods**

**SREBP2 activation by recombinant SARS-CoV-2 Spike proteins in HUVECs** Recombinant SARS-CoV-2 Spike RBD His-tag Protein (10534-CV-100, R&D systems, Minneapolis, MN) were diluted in DMEM Medium to treat in vitro HUVECs cultures (2×10^5^ cells/flask). To simulate a virus–cell interaction, cultures were washed twice in DMEM, treated with various doses of S protein, (25 or 50 nM) in DMEM per 25cm^2^/flask at 37 °C. After 24 h, nuclear cell fractions were assayed for active SREBP2 transcription factor. All RNA-seq data were mapped using the Tophat package.

**Inflammatory cytokines IL-1β, and TNF-α ELISAs**

Serum levels of inflammatory cytokines IL-1β, and TNF-α were determined in SARS-CoV-2-infected patients using human ELISA kits (Quantikine ELISA, R&D Systems, Minneapolis, MN, USA) according to the manufacturer’s instructions. The results were expressed as pg/mL.

**WST-1 cell proliferation assay**

10 μL per well of WST-1 reagent were added in purified PBMC and incubated at 37 °C with 5% CO_2_. At indicated time points, measurements of absorbance were taken at 480 nm and 600 nm (background) on Tecan Spark microplate reader.

**Real-time PCR**

To generate cDNA from PBMCs, SN50/Fatostatin A treated PBMCs, or SN50/Fatostatin A administrated septic mouse lung tissue samples, 1 μg of total RNA was reverse transcribed with random hexamers using expand reverse transcription polymerase (Roche). Real-time PCR was performed using the LightCycler FastStart DNA Master SYBR Green I from Roche Diagnostics GmbH according to the manufacturer’s protocol. The following LightCycler conditions were used: Initial denaturation at 95°C for 10 min, followed by 45 cycles with denaturation at 95°C for 10 min, annealing at 60°C for 5 min, and elongation at 72°C for 15 min. Quantities of specific mRNA in the sample were measured according to the corresponding gene-specific standard curves. The primer sequences are in the **Supplemental Table 2.**

**Laboratory tests**

CRP and LDH were determined using a Roche/Hitachi Modular DP Chemistry Analyzer.

**Western blot**

SREBP-2 in LPS-treated HUVECs were detected by immunoblotting. After SDS-PAGE, we performed an immunoblotting assay with each antibody. Anti-SREBP-2 N-term antibody (MABS1988, Millipore), anti-SREBP-2 C-term antibody (ab30682, Abcam), anti-β-actin antibody (sc-47778, Santa Cruz), anti-ABCA1 antibody (sc-53482, Santa Cruz).

**Cholesterol staining by filipin**

HUVEC were seeded in a Nunc Lab-Tek II 8-Chamber Slide and treated with compounds for indicated time points. Cells were fixed with 4% paraformaldehyde for 1 hour at room temperature and then permeabilized with 0.5% Triton X-100 (for protein immunostaining) for 10min prior to blocking in a blocking buffer (3% bovine serum albumin (BSA) in PBS containing 0.1% Tween-20) for 12h. Cells were incubated with Filipin statining dye in the blocking buffer overnight at 4°C. Cells were washed with PBS, mounted with Immu-mount, and were visualized by fluorescence microscopy at a 200× magnification (Leica microsystem, Germany).

**RNA analysis**

RNA from rhControl + LPS treated HUVECs or rhSREBP2 + LPS treated HUVECs was extracted using RNeasy mini-kit (Qiagen Venlo, Netherlands) according to the manufacturer’s protocols. RNA-seq libraries were prepared using the TruSeq RNA Sample Prep kit v2 (Illumina) according to the manufacturer’s protocols. RNA-seq libraries were pair-end sequenced on an Illumina Hi-seq 3000/4000 SBS kit v3 (MACROGEN Inc.).[^1^](#_ENREF_1) against Affymetrix Human Gene 2.0 ST arrays (902136). Remaining mRNA was used for qPCR analysis. Fold-change was determined using the R package limma, and P-values were Benjamini-Hochberg (BH) adjusted. The array results are available in the Gene Expression Omnibus (GEO) database of NCBI (Accession code: GSE101126).

**In vitro permeability assay**

For spectrophotometric quantification of endothelial cell permeabilities in response to the increasing concentrations of each molecule, the flux of Evans blue-bound albumin across functional cell monolayers was measured using a modified 2-compartment chamber model, as previously described.[^2^](#_ENREF_2) HUVECs were plated (5 × 10^4^/well) for 3 days in 12-mm diameter Transwells with a pore size of 3 µm. Confluent monolayers of HUVECs, SREBP-2 knockdown by shRNA, or overexpressed HUVECs were exposed to LPS (100 ng/mL) for 6 h before being exposed to SN50, FatostatinA, or PF-429242. Transwell inserts were then washed with TBS (pH 7.4), followed by the addition of Evans blue (0.5 mL; 0.67 mg/mL) diluted in a growth medium containing 4% BSA. Fresh growth medium was then added to the lower chamber and the medium in the upper chamber was replaced with Evans blue/BSA. Ten minutes later, the optical density of the sample in the lower chamber was measured at 650 nm.

**Animals**

Animal experiments were carried out in accordance with protocols approved by the Institutional Animal Care and Use Committee (IACUC) of Kyungpook National University (IACUC-No. KNU 2017-102). Six- to seven-week-old C57BL/6 male mice (18–20 g) were obtained from Orient Bio (Seongnam, Korea). Mice were used after a 12-day acclimatization period. Five animals per cage were housed under controlled temperature at 20–25 °C and humidity of 40–45% with a 12:12 h light/dark cycle. Mice were fed a normal rodent pellet diet and supplied with water *ad libitum*.

**Cecal ligation and puncture**

The CLP-operated septic mouse model was prepared as previously described.[^3^](#_ENREF_3) Briefly, a 2-cm midline incision was made to expose the cecum and adjoining intestine. The cecum was then ligated tightly using a 3.0-silk suture 5.0 mm from the cecal tip, punctured with a 22-gauge needle, and then gently squeezed to extrude feces from the perforation site. The cecum was then returned to the peritoneal cavity, and the laparotomy site was sutured using 4.0-silk. For sham operations, the cecum of animals was surgically exposed, but not ligated or punctured, and then returned to the abdominal cavity.

**Hematoxylin & eosin (H&E) staining and histopathological examination**

Male C57BL/6 mice were CLP-operated and then intravenously administered SN50 (dose) or FatoA at 24 h or 48 h after CLP (n = 5). SREBP-2 Knockdown mice were transfected SREBP-2 shRNA complex with PEG-Liposome *In Vivo* Transfection Kit (Altogen biosystem). At 72 h post-CLP operation, mice were euthanized. Lung specimens were removed from mice for the analysis of phenotypic changes. H&E staining was performed using a standard protocol.

**Cytokine levels in the plasma of septic mice**

Fresh serum was used for analysis of AST, ALT, BUN, creatinine, and LDH levels using biochemical kits (MyBioSource). Values were measured using an ELISA plate reader (Tecan, Austria GmbH, Austria).

**Reference**

1. Kim, D. *et al.* TopHat2: accurate alignment of transcriptomes in the presence of insertions, deletions and gene fusions. *Genome Biol.* **14**, R36 (2013).

2. Lee, W. *et al.* A Double-Chambered Protein Nanocage Loaded with Thrombin Receptor Agonist Peptide (TRAP) and gamma-Carboxyglutamic Acid of Protein C (PC-Gla) for Sepsis Treatment. *Adv. Mater.* **27**, 6637-6643 (2015).

3. Rittirsch, D., Huber-Lang, M.S., Flierl, M.A. & Ward, P.A. Immunodesign of experimental sepsis by cecal ligation and puncture. *Nat. Protoc.* **4**, 31-36 (2009).


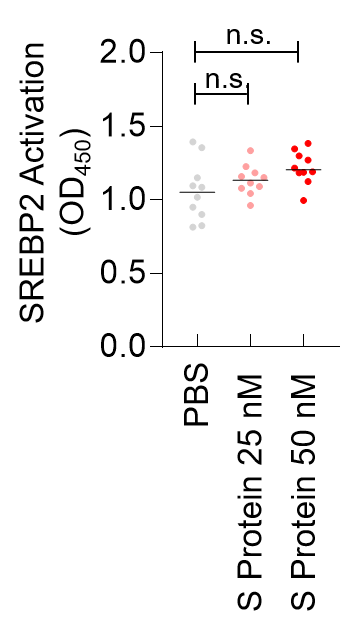


**Figure. S1.** Recombinant SARS-CoV-2 spike RBD protein mediated SREBP2 activation in HUVECs (n.s*.*: not significant).


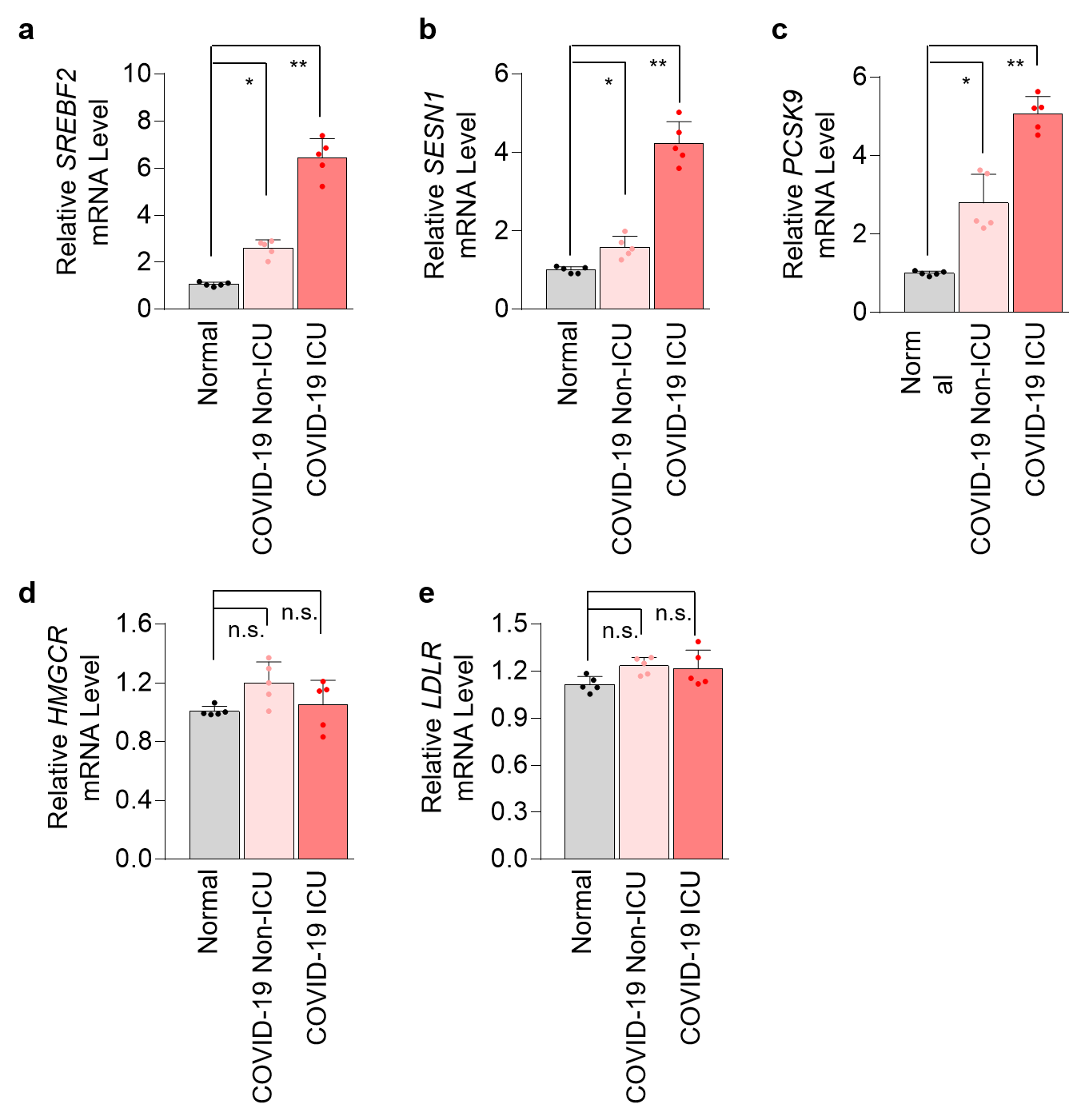


**Figure. S2.** Relative mRNA level of SREBF2 (a), SESN1 (b), PCSK9 (c), HMGCR (d), and LDLR (e) (*p<0.05, **p<0.01, n.s.: not significant).


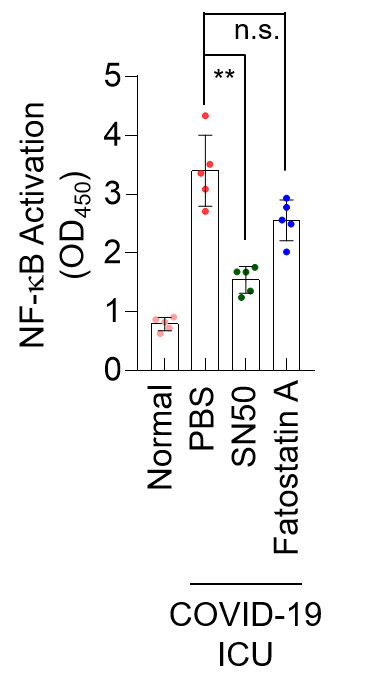


**Figure. S3.** Level of NF-κB activation in COVID-19 ICU patients’ PBMCs by the treatment of SN50 and Fatostatin A (*p<0.05, **p<0.01, n.s.: not significant).


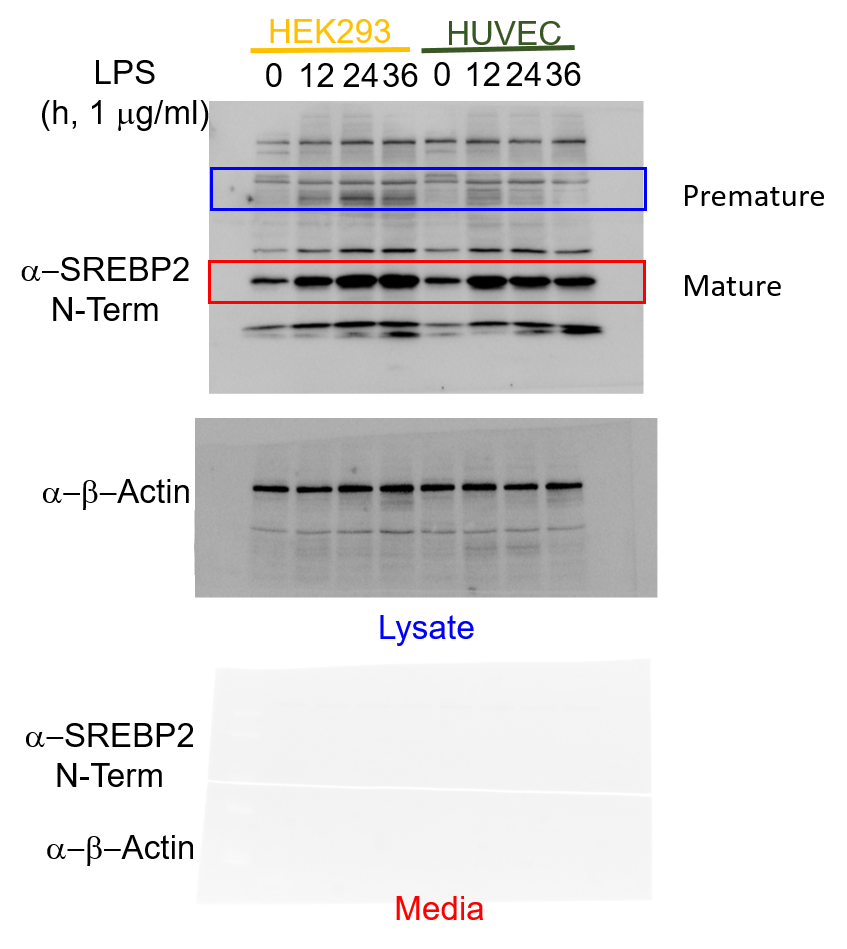


**Figure. S4.** Western blot analysis of SREBP-2 N-term in lysate and media of HEK293 and HUVEC.


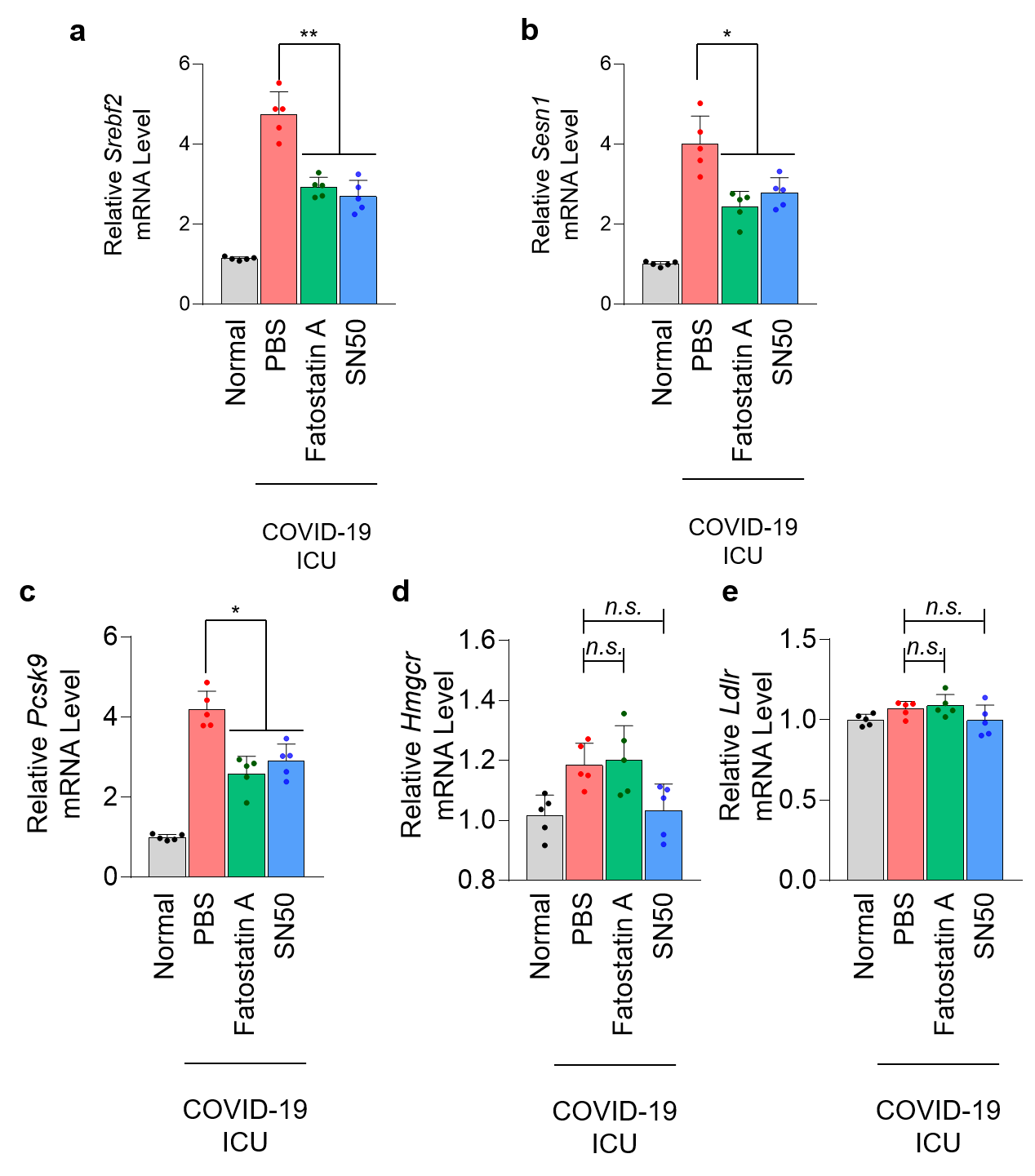


**Figure. S5.** Relative mRNA expression levels of Srebf2 (a), Sesn1(b), Pcsk9 (c), Hmgcr (d), and Ldlr (e) (*p<0.05, **p<0.01, n.s.: not significant).


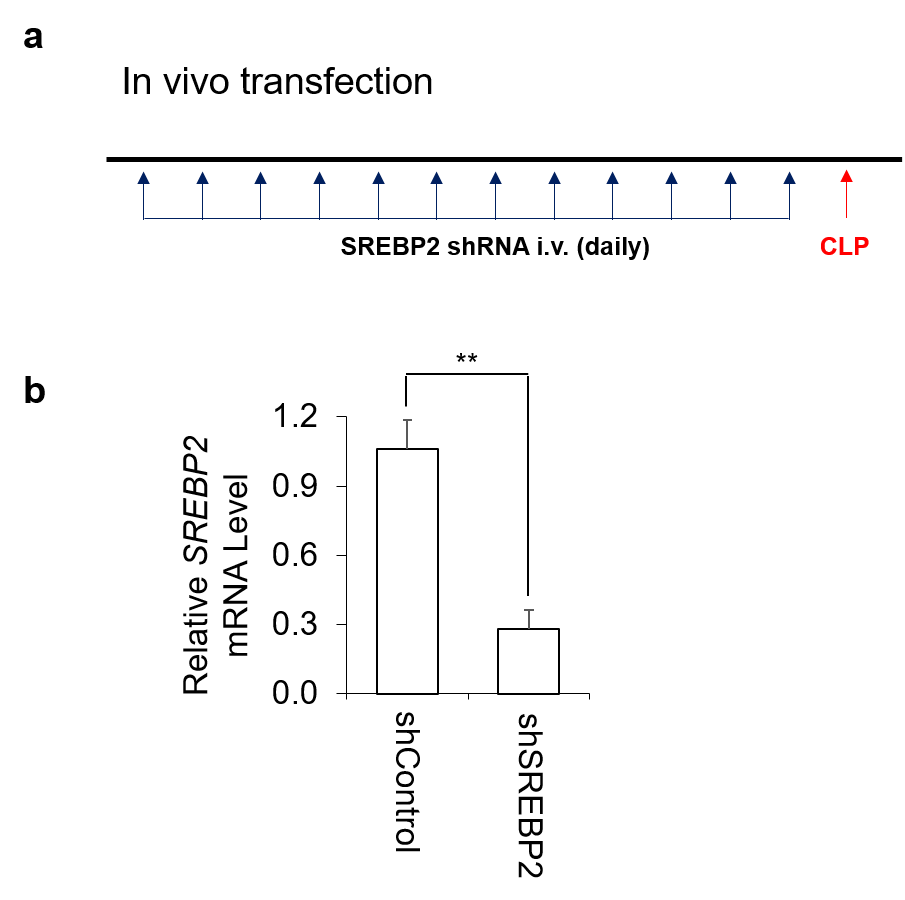


**Figure. S6.** (a) The time table of shRNA mediated SREBP-2 genetic ablation. (b) Relative mRNA level of SREBP-2 after shSREBP-2 treatment. (n.s.: not significant)


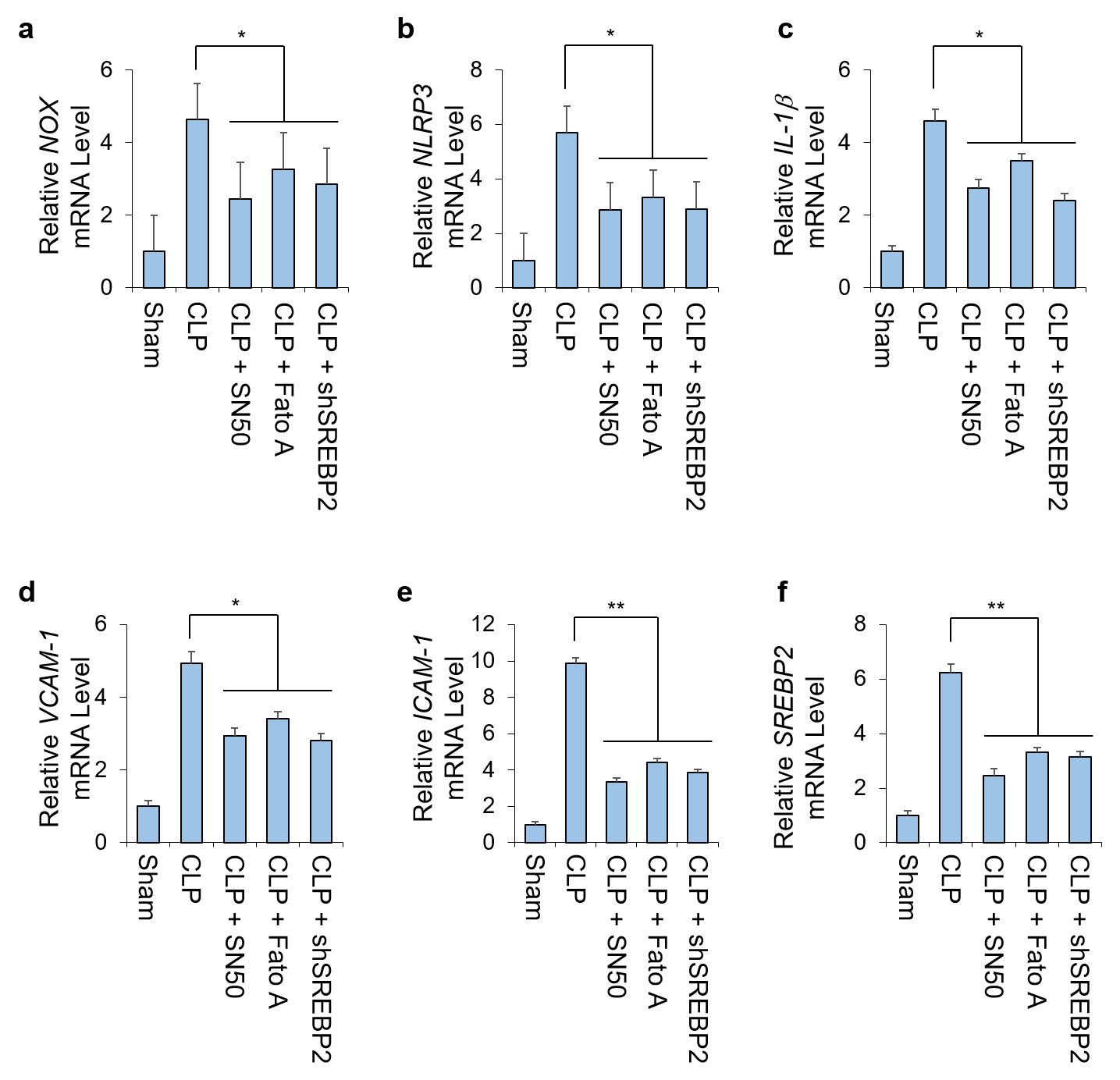


**Figure. S7.** Relative mRNA levels of NOX (a), NLRP3 (b), IL-1β (c), VCAM-1 (d), ICAM-1 (e), and SREBP-2 (f) in mouse lung tissue (*p<0.05, **p<0.01, n.s.: not significant).


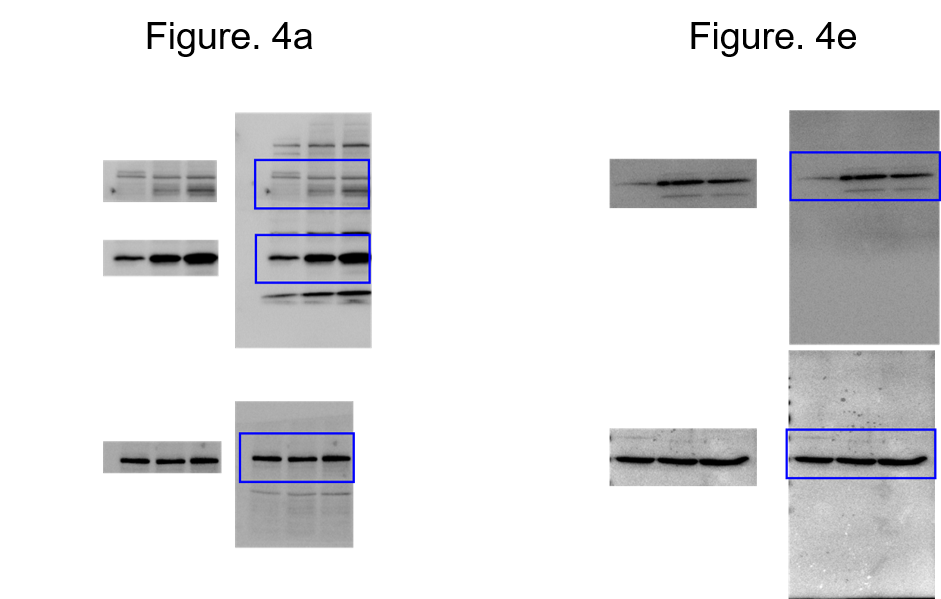


**Figure. S8.** Full size image of western blot data

**Table S1.** **Baseline characteristics and clinical outcomes of COVID-19 patients admitted to Yeungnam University Hospital.**

|  | Normal  (n=20) | Non-ICU  (n = 50) | ICU  (n = 20) | Normal Range |
| --- | --- | --- | --- | --- |
| **Characteristics** |  |  |  |  |
| Age, y | 32.5 ± 11.8 | 46.8.2 ± 21.1 | 74.8 ± 10.5 |  |
| Sex |  |  |  |  |
| Men | 10(50) | 25 (50) | 8 (40) |  |
| Women | 10(50) | 25 (50) | 12 (60) |  |
| Serum glucose (mg/dL) | 108.6 ± 23.5 | 113.6 ± 31.8 | (non-DM)  72.8 ± 26.5  (DM)  192.5 ± 48.7 | 70-140 mg/dL |
| **Comorbidities** |  |  |  |  |
| Cardiovascular disease | 0 (0) | 0 (0) | 0 (0) |  |
| Cerebrovascular disease | 0 (0) | 0 (0) | 0 (0) |  |
| ^*^Chronic lung disease^*^ | 0 (0) | 0 (0) | 0 (0) |  |
| Dementia | 0 (0) | 0 (0) | 0 (0) |  |
| Diabetes mellitus | 0 (0) | 0 (0) | 0 (0) |  |
| Hypertension | 0 (0) | 0 (0) | 0 (0) |  |
| Liver disease | 0 (0) | 0 (0) | 0 (0) |  |
| Malignancy | 0 (0) | 0 (0) | 0 (0) |  |
| Parkinson’s disease | 0 (0) | 0 (0) | 0 (0) |  |
| **Cholesterol profiles** | |  |  |  |
| Total Cholesterol (mg/dL) | 211.2 ± 20.7 | 186.5 ± 13.8 | 142.8 ± 26.5 | 200-239 mg/dL |
| HDL-Ch (mg/dL) | 67.5 ± 7.2 | 60.6 ± 10.4 | 42.5 ± 7.2 | 60 mg/dL |
| LDL-Ch (mg/dL) | 108.3 ± 12.5 | 96.2 ± 11.8 | 69.5 ± 9.4 | 100-129 mg/dL |
| **Clinical outcomes** |  |  |  |  |
| Remained in hospital | 0 (0) | 10 (20) | 11 (55) |  |
| Discharged | 0 (0) | 40 (80) | 0 (0) |  |
| Died | 0 (0) | 0 (0) | 9 (45) |  |

Data are presented as mean ± SD (range) or number (percentage).

^*^ Chronic lung disease includes COPD, asthma, bronchiectasis, and interstitial lung disease.

ICU = intensive care unit.

**Table S2. Real-time PCR Primer Sequences**

| **Gene symbol** | **Accession number** | **Forward primer** | **Reverse primer** |
| --- | --- | --- | --- |
| hSREBF2 | [NM_004599.3](http://www.ncbi.nlm.nih.gov/nuccore/NM_004599.3) | CCTTCCTGTGCCTCTCCTTTA | AGGCATCATCCAGTCAAACCA |
| hSESN1 | [NM_014454.2](http://www.ncbi.nlm.nih.gov/nuccore/NM_014454.2) | GGCTGAAGGAGAGAATGAAGTG | CTGATGGACGATGAGGTGTTTC |
| hPCSK9 | [NM_174936.3](http://www.ncbi.nlm.nih.gov/nuccore/NM_174936.3) | GGACTTTGGGGACCAACTTTG | GTGGATCAGTCTCTGCCTCAA |
| hHMGCR | [NM_000859.2](http://www.ncbi.nlm.nih.gov/nuccore/NM_000859.2) | TGGTGATGGGAGCTTGTTGTG | GCACCTCCACCAAGACCTATT |
| hLDLR | [NM_001195803.1](http://www.ncbi.nlm.nih.gov/nuccore/NM_001195803.1) | TCACCAAGCTCTGGGCGACG | GTAGCCGTCCTGGTTGTGGCA |
| hIL1B | [NM_000576.2](http://www.ncbi.nlm.nih.gov/nuccore/NM_000576.2) | TACCTGTCCTGCGTGTTGAAA | CTGCTTGAGAGGTGCTGATGT |
| hTNF | [NM_000594.3](http://www.ncbi.nlm.nih.gov/nuccore/NM_000594.3) | TGGCGTGGAGCTGAGAGATAA | TTGATGGCAGAGAGGAGGTTGA |
| hSCAP | [NM_012235.2](http://www.ncbi.nlm.nih.gov/nuccore/NM_012235.2) | GCGAGATTTTCCCCTACCTTGT | CCACGACAGCAAAGAGACAGA |
| hINSIG1 | [NM_005542.4](http://www.ncbi.nlm.nih.gov/nuccore/NM_005542.4) | TTTGGGCCTTTGGTGGACATT | ACTGTCGTCCTATGTTCCCCA |
| hSIRT1 | [NM_012238.4](http://www.ncbi.nlm.nih.gov/nuccore/NM_012238.4) | TTGGCACAGATCCTCGAACAA | ACACCCCAGCTCCAGTTAGAA |
| mSrebf2 | [NM_033218.1](http://www.ncbi.nlm.nih.gov/nuccore/NM_033218.1) | TGTGGCTGGTAAATGGTGTGA | AGCACGGATAAGCAGGTTTGT |
| mSesn1 | [NM_001162908.1](http://www.ncbi.nlm.nih.gov/nuccore/NM_001162908.1) | GACACTTTCCCGACCTTCAGA | CGCTTCTCTCTCGCACATCTT |
| mPcsk9 | [NM_153565.2](http://www.ncbi.nlm.nih.gov/nuccore/NM_153565.2) | ATTGTGGTGCTGATGGAGGAG | AGAAGCCAGGGAAGAGGTCAT |
| mHmgcr | [NM_008255.2](http://www.ncbi.nlm.nih.gov/nuccore/NM_008255.2) | CGAGCCACGACCTAATGAAGA | GGCGAATAGACACACCACGTT |
| mLdlr | [NM_010700.3](http://www.ncbi.nlm.nih.gov/nuccore/NM_010700.3) | GGCCGTCTCTATTGGGTTGAT | TTGGCACTGAAAATGGCTTCG |
| mNox | [NM_010700.3](http://www.ncbi.nlm.nih.gov/nuccore/NM_010700.3) | GGTTGGGGCTGAACATTTTTC | TCGACACACAGGAATCAGGAT |
| mNlrp3 | [NM_145827.3](http://www.ncbi.nlm.nih.gov/nuccore/NM_145827.3) | TCGCCCAAGGAGGAAGAAGAA | TGAGAAGAGACCACGGCAGAA |
| mIl-1β | [NM_008361.4](http://www.ncbi.nlm.nih.gov/nuccore/NM_008361.4) | GGTGTGTGACGTTCCCATTAG | TCGTTGCTTGGTTCTCCTTGT |
| mVcam-1 | [NM_011693.3](http://www.ncbi.nlm.nih.gov/nuccore/NM_011693.3) | GGAAATGCCACCCTCACCTTA | ACAACCGAATCCCCAACTTGT |
| mIcam-1 | [NM_010493.2](http://www.ncbi.nlm.nih.gov/nuccore/NM_010493.2) | GCTACCATCACCGTGTATTCG | TAGCCAGCACCGTGAATGTG |
| h β -actin | [NM_007393.5](http://www.ncbi.nlm.nih.gov/nuccore/NM_007393.5) | CATGTACGTTGCTATCCAGGC | CTCCTTAATGTCACGCACGAT |
| mGapdh | [NM_001289726.1](http://www.ncbi.nlm.nih.gov/nuccore/NM_001289726.1) | AGGCCGGTGCTGAGTATGTC | TGCCTGCTTCACCACCTTCT |
